# A meta-analysis of the efficacy of limus-coated balloons vs. paclitaxel-coated balloons for coronary artery disease

**DOI:** 10.3389/fcvm.2026.1872397

**Published:** 2026-07-14

**Authors:** Shichuan Li, Sheng Guo, Yangbo Deng, Yuying Gao, Yinmei Xiong

**Affiliations:** 1 Department of Cardiology, Chongqing Rongchang District People's Hospital, Chongqing, China; 2 Department of Pediatrics, Chongqing Rongchang District People’s Hospital, Chongqing, China

**Keywords:** coronary artery disease, drug-coated balloon, limus, meta-analysis, paclitaxel

## Abstract

**Objective:**

To evaluate the efficacy of limus-coated balloons (Limus-DCB) compared with paclitaxel-coated balloons (PCB) in the treatment of coronary artery disease.

**Methods:**

We searched PubMed, Embase, Cochrane Library, and Web of Science for randomized controlled trials (RCTs) comparing Limus-DCB with PCB for coronary artery disease from inception to June 2026. Two reviewers independently screened the literature, extracted data, and assessed the risk of bias of the included studies. Meta-analysis was performed using RevMan 5.4.1 software.

**Results:**

A total of 10 studies involving 11 RCTs with 1,707 patients and 1,804 treated vessels were included. The meta-analysis showed that the Limus-DCB group was associated with marginally greater in-segment late lumen loss (LLL) (MD = 0.10, 95% CI 0.00–0.19, *P* = 0.04) and marginally smaller in-lesion minimal lumen diameter (MLD) (MD = −0.10, 95% CI −0.19 to 0.00, *P* = 0.04). There were no significant differences between the two groups in in-lesion LLL, late lumen enlargement (LLE), binary restenosis rate, target lesion failure (TLF), or major adverse cardiovascular events (MACE).

**Conclusions:**

Based on current evidence, compared with PCB, Limus-DCB are associated with less favorable angiographic outcomes in the treatment of coronary artery disease. However, no statistically significant short-term clinical differences were detected between the two groups. Due to the limitations in the quantity and quality of the included studies, these findings need to be verified by more high-quality research.

**Systematic Review Registration:**

https://www.crd.york.ac.uk/PROSPERO/recorddashboard, identifier CRD420251153751.

## 1 Introduction

With continuous improvements in devices, the therapeutic approach to percutaneous coronary intervention (PCI) has evolved progressively. From initial plain old balloon angioplasty (POBA) to bare-metal stent (BMS) implantation, and subsequently to the clinical application of drug-eluting stents (DES), the efficacy and prognosis of interventional therapy have been significantly enhanced. However, stent implantation is also associated with certain issues, such as in-stent restenosis (ISR), delayed vascular endothelial healing, late stent malapposition, and stent thrombosis (1). Consequently, the drug-coated balloon (DCB), embodying the novel concept of “intervention without implantation,” has emerged as an alternative technology. DCB combines conventional balloon angioplasty with drug elution technology, whereby anti-proliferative drugs are attached to the balloon surface. During inflation, the drug is transferred from the balloon to the local vessel wall, thereby inhibiting smooth muscle cell proliferation and preventing vascular restenosis. Paclitaxel-coated balloons (PCB) have long been the cornerstone of DCB therapy due to their well-established clinical efficacy (2–4). However, sirolimus-coated balloons (SCB) have demonstrated reduced re-endothelialization and inflammatory infiltration in animal experiments and optical coherence tomography studies (5, 6), potentially lowering long-term complications. This study systematically evaluates the efficacy of Limus-DCB compared with PCB for treating coronary artery disease, aiming to provide evidence for clinical practice.

## 2 Methods

The conduct of this review adhered to the Cochrane Methodological Expectations for Intervention Reviews (MECIR) (7), and its reporting strictly followed the guidelines outlined in the Preferred Reporting Items for Systematic Reviews and Meta-Analyses (PRISMA) statement (8).

### 2.1 Types of studies, eligibility criteria and interventions

The type of studies included in this review were randomized controlled trials (RCTs). The study subjects were patients with a definite diagnosis of coronary artery disease requiring interventional treatment. The intervention in the experimental group was Limus-DCB, and that in the control group was PCB. Conference abstracts were excluded because they lack sufficient methodological detail for quality assessment and may present preliminary data subject to change in subsequent full-text publications.

### 2.2 Outcome measures

1. Angiographic outcome indicators included: late lumen loss (LLL), minimal lumen diameter (MLD), late lumen enlargement (LLE), and binary restenosis rate (defined as angiographic stenosis ≥50%). In-segment was defined as the treated area plus 5 mm proximally and distally, while in-lesion referred to the treated area only.
2. Clinical outcomes included: procedural success, target lesion failure (TLF), target lesion revascularization (TLR), thrombosis, all-cause mortality, cardiac mortality, myocardial infarction (MI), target vessel myocardial infarction (TVMI), and major adverse cardiovascular events (MACE). Definitions of outcome measures reported in the included studies are shown in Supplementary Table S1.

### 2.3 Literature search, data collection and assessment

A computerized search was conducted in PubMed, Embase, Cochrane Library, and Web of Science to collect RCTs comparing Limus-DCB with PCB for coronary artery disease, with the search period spanning from database inception to June 2026. Furthermore, the references of included studies were manually searched to supplement relevant literature. The search used a combination of MeSH terms and free words. English search terms included “Drug-Coated Balloon” “Sirolimus” “Rapamycin” “limus” “biolimus” “Randomized Controlled Trial”, etc. Search Strategies are shown in Supplementary Table S2. After completing the literature search, two reviewers first independently screened titles and abstracts against the predefined criteria, excluding irrelevant records. Subsequently, the full-text articles of the remaining potentially eligible studies were obtained and subjected to further assessment. All procedures, including the search strategy implementation, article screening, and data extraction, were conducted independently by two reviewers. The data extraction encompassed study design, intervention protocols, sample sizes per group, demographic profiles, eligibility criteria, endpoints, along with parameters relevant to methodological quality evaluation. Disagreements between reviewers were addressed through discussion, with arbitration by a third reviewer when consensus could not be reached.

### 2.4 Risk of bias

We utilized the Cochrane Risk of Bias Tool (RoB 2) (9) to assess the quality of the included RCTs, which encompasses five key domains: randomization process, deviations from intended interventions, missing outcome data, measurement of the outcome, and selection of the reported result. According to the RoB 2 tool, each domain was judged as “low risk of bias,” “some concerns,” "or “high risk of bias.” The overall risk of bias for each trial was then summarized based on these domain-level judgments. Two researchers independently assessed the risk of bias for each study. Disagreements between reviewers were addressed through discussion, with arbitration by a third reviewer when consensus could not be reached.

### 2.5 Statistical analysis

RevMan 5.4.1 software was used for meta-analysis. Mean difference (MD) was used as the effect indicator for continuous data, and odds ratio (OR) was used for dichotomous data. Each effect size was presented with its 95% confidence interval (CI). Due to differences in lesion types, DCB, and vessel diameters across studies, a random-effects model was used for meta-analysis. Heterogeneity was assessed using the *I*^2^ statistic, interpreted according to Cochrane guidelines as: 0%–40% (might not be important); 30%–60% (moderate); 50%–90% (substantial); 75%–100% (considerable) (10). Subgroup analysis was performed for significant clinical heterogeneity, following the same interpretation framework. If meta-analysis was not possible, a descriptive analysis was performed. For outcomes with substantial heterogeneity (*I*^2^ > 50%) and at least five studies, we calculated 95% prediction intervals (PI) using the Cochrane-recommended t-distribution method based on *τ*^2^ and SE estimates derived from the random-effects model in RevMan 5.4.1. We used a fixed-effects model to assess the robustness of the synthesized results. For outcome measures, intention-to-treat (ITT) data were preferentially used when available. In cases where a study reported only per-protocol results without providing ITT data, the per-protocol data were included. Angiographic outcomes were analyzed at the vessel level (number of treated vessels), while clinical outcomes were analyzed at the patient level (number of patients).

## 3 Results

### 3.1 Literature search

**Figure 1:**
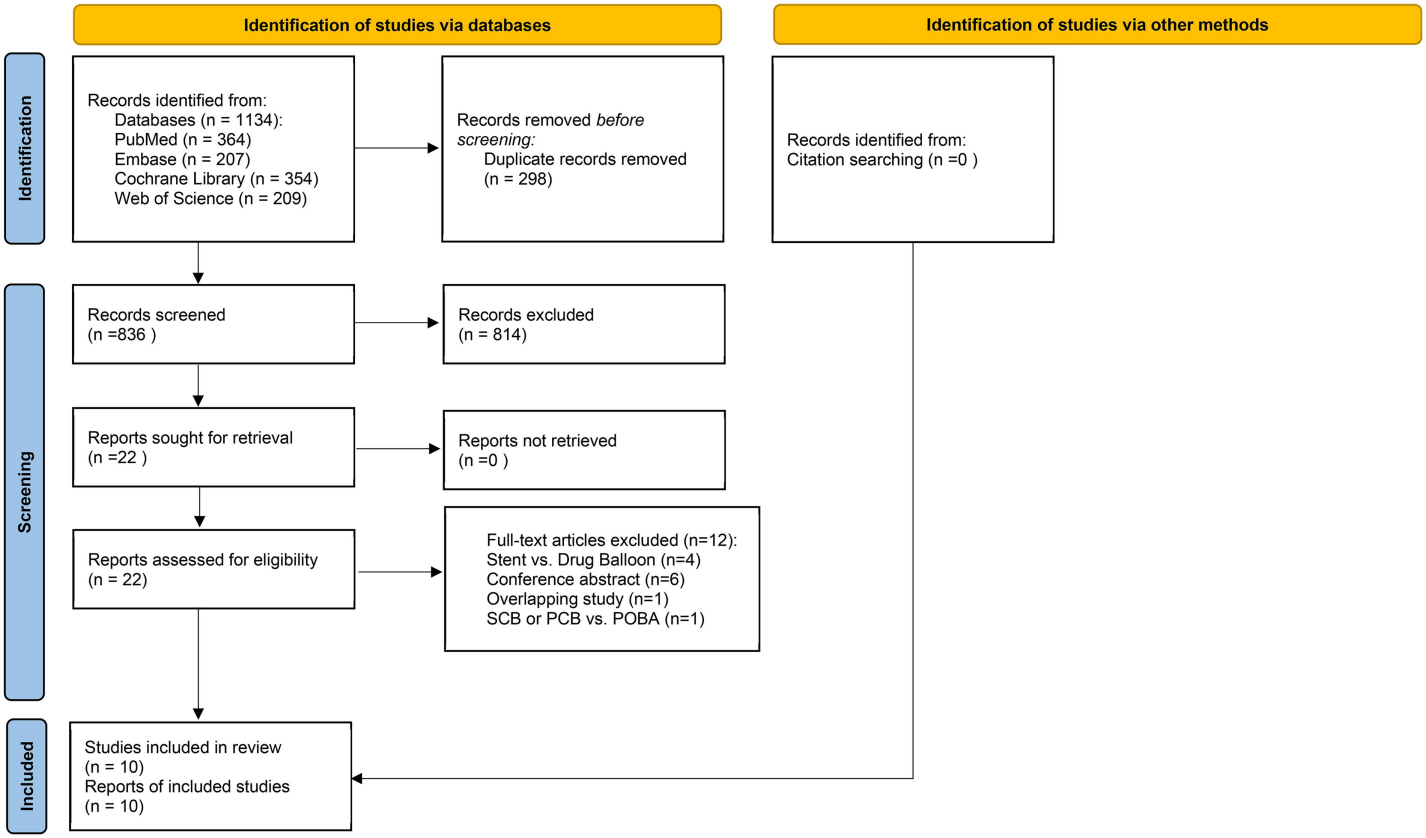
Literature screening flow diagram.

A total of 1,134 relevant articles were initially identified. After sequential screening, 11 RCTs (6, 11–20) met the inclusion criteria. However, one study [Ali et al. (20)] was excluded from the quantitative analysis because its patient population had already been incorporated into the pooled analysis of Scheller et al. (18); including both would have resulted in double-counting of the same patients. Accordingly, 10 studies (6, 11–19) (comprising data from 11 RCTs) were included in the final meta-analysis, comprising 1,707 patients and 1,804 treated vessels. The reasons for excluding full-text articles are provided in Supplementary Table S3. The literature screening process and results are shown in Figure 1.

### 3.2 Study characteristics and quality assessment

The included studies varied in lesion types, the investigational drug-coated balloons used in the experimental groups, and analytical methods. Five studies (11–13, 17, 19) included patients with coronary *de novo* lesions, one of two (17, 19) involved bifurcation lesions, while the remaining five studies (6, 14–16, 18) included patients with ISR. In the PCB group, except for two studies (17, 19) that used Bingo (Yinyi Biotech), all others used SeQuent Please Neo (B. Braun). The Limus-DCB used varied by study: five studies (6, 11, 13, 17, 18) used the SeQuent Neo (B. Braun) SCB, two studies (12, 14) used the MagicTouch (Concept Medical) SCB, one study (19) used Revita Medical Technology SCB, one study (15) used the BioAscend JWMS biolimus-coated balloon (BCB), and one study (16) used the Biosensors Europe BCB. Regarding follow-up duration, coronary angiography follow-up periods ranged from 6 to 12 months, and follow-up for clinical outcome measures ranged from 9 to 12 months. Except for one study (14) that included patients with acute myocardial infarction, all other trials excluded patients within 3 days of myocardial infarction. Reference vessel diameters ranged from 2.0 to 5.0 mm across the included studies. Among the included studies, except for one study (12) which only provided per-protocol data, the data for the pooled analysis were all from intention-to-treat populations. The basic characteristics of the included studies are shown in Table 1.

**Table 1:** Baseline characteristics and details of the included studies.

**Included study**	**Region**	**Patients (T/C, n)**	**Vessels (T/C, n)**	**Age (T/C, years)**	**Lesion type**	**Vessel diameter (mm)**	**Experimental balloon**	**Angiographic follow-up duration**	**Clinical follow-up duration**	**Outcome measures**
Liu (6)	China	130/128	149/141	63.8 ± 9.3/ 63.7 ± 8.5	ISR	2.5–4.0	SCB	9 m	12 m	①②④⑤
Scheller (11)	Germany/Switzerland	35/35	39/37	66 ± 9/67 ± 12^a^	*de novo*	≥2.5	SCB	6 m	12 m	①②③④⑤
Ninomiya (12)	Europe	61/60	66/63	70 (63–74)/67 (59–72)^b^	*de novo*	2.0–2.75	SCB	6 m	10 m	①②③④⑤
Ahmad (13)	Malaysia	35/35	37/38	60 ± 11/59 ± 12	*de novo*	≥2.5	SCB	6 m	12 m	①②③④⑤
Pleva (14)	Czech Republic	72/73	79/79	69.7 ± 9.6/68.0 ± 10.6	ISR	2.25–5.0	SCB	12 m	12 m	①④⑤
Chen (15)	China	138/137	152/153	63.64 ± 8.90/64.24 ± 8.84	ISR	2.79 ± 0.40/2.80 ± 0.39	BCB	9 m	12 m	①②③④⑤
Byrne (16)	Europe/Asia	135/67	135/67	68.9 ± 9.8/68.3 ± 10.4	ISR	≥2.5	BCB	6 m	12 m	①②④⑤
Zhou (17)	China	114/115	114/115	66 (60–71)/64 (57–69)	*de novo*	2.0–2.5	SCB	9 m	9 m	④⑤
Scheller (18)^c^	Germany/Switzerland/Malaysia	50/51	52/52	67 ± 12/63 ± 12	ISR	≥2.5	SCB	6 m	12 m	①②④⑤
Gao (19)	China	119/117	119/117	63.92 ± 9.48/61.51 ± 9.42	*de novo*	2.0–4.0	SCB	9 m	12 m	①④⑤

T, experimental group; C, control group; ISR, in-stent restenosis; SCB, sirolimus-coated balloon; BCB, biolimus-coated balloon; m, months. ① late lumen loss (LLL); ② minimal lumen diameter (MLD); ③ late lumen enlargement (LLE); ④ binary restenosis rate; ⑤ clinical outcomes.

a mean ± standard deviation.

b median (interquartile range).

c pooled analysis.

The results of the risk of bias assessment showed that the randomization process was rated as low risk in all 10 included studies, as each study was clearly described as a randomized controlled design, with most using computer-generated or centrally allocated randomization sequences, some employing allocation concealment measures such as sealed envelopes, and with balanced baseline characteristics between groups. All 10 studies were rated as having a high risk of bias due to the open-label design, which made it impossible to blind the operators (high risk of performance bias). All 10 studies blinded the outcome assessors (core laboratory and/or clinical events committee). Regarding the completeness of outcome data, four studies (6, 15, 17, 19) validated the robustness of their results through FAS/PPS dual analyses [with Gao et al. (19) additionally performing worst-case imputation and predictive mean matching sensitivity analyses]; four studies (12–14, 18) were rated as low risk due to low loss to follow-up (<10%) and balanced distribution between groups. Scheller et al. (11) was rated as having some concerns due to a notable imbalance in loss to follow-up between the two groups (SCB 89% vs. PCB 80%). Byrne et al. (16) was rated as having some concerns for the angiographic outcome due to the use of available-case analysis without imputation or sensitivity analysis for missing data, leaving the possibility that missingness was related to the true outcomes. The risk of bias assessment results are shown in Table 2.

**Table 2:** Risk of bias assessment for the included studies.

**Included study**	**Randomization process**	**Deviations from intended intervention**	**Missing outcome data**	**Measurement of the outcome**	**Selective reporting**	**Overall risk**
Liu (6)	Low	High	Low	Low	Low	High
Scheller (11)	Low	High	Some concerns	Low	Low	High
Ninomiya (12)	Low	High	Low	Low	Low	High
Ahmad (13)	Low	High	Low	Low	Low	High
Pleva (14)	Low	High	Low	Low	Low	High
Chen (15)	Low	High	Low	Low	Low	High
Byrne (16)	Low	High	Some concerns	Low	Low	High
Zhou (17)	Low	High	Low	Low	Low	High
Scheller 18)	Low	High	Low	Low	Low	High
Gao (19)	Low	High	Low	Low	Low	High

### 3.3 Meta-analysis results

#### 3.3.1 Angiographic outcome indicators

**Figure 2:**
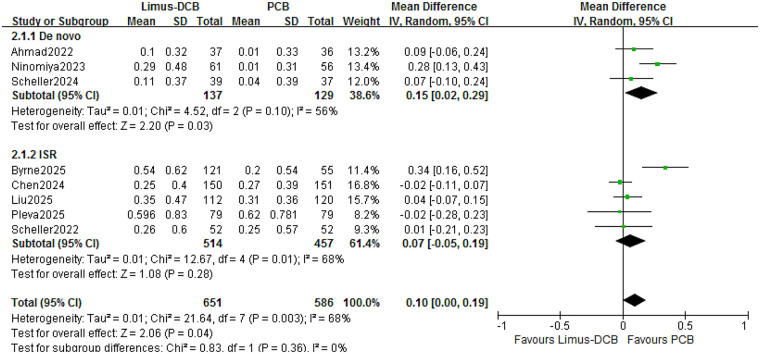
Forest plot of in-segment LLL: limus-DCB versus PCB.

**Figure 3:**
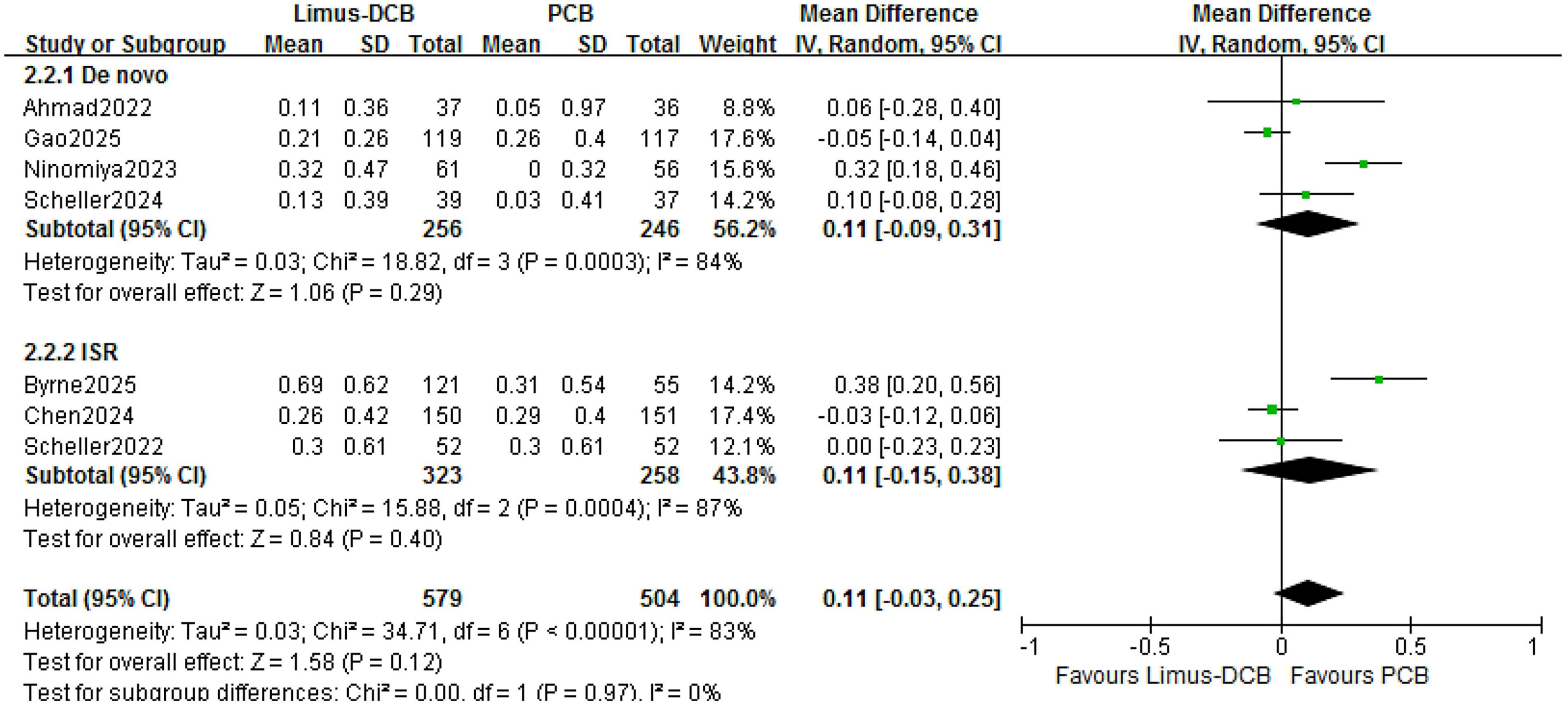
Forest plot of in-lesion LLL: limus-DCB versus PCB.

**Figure 4:**
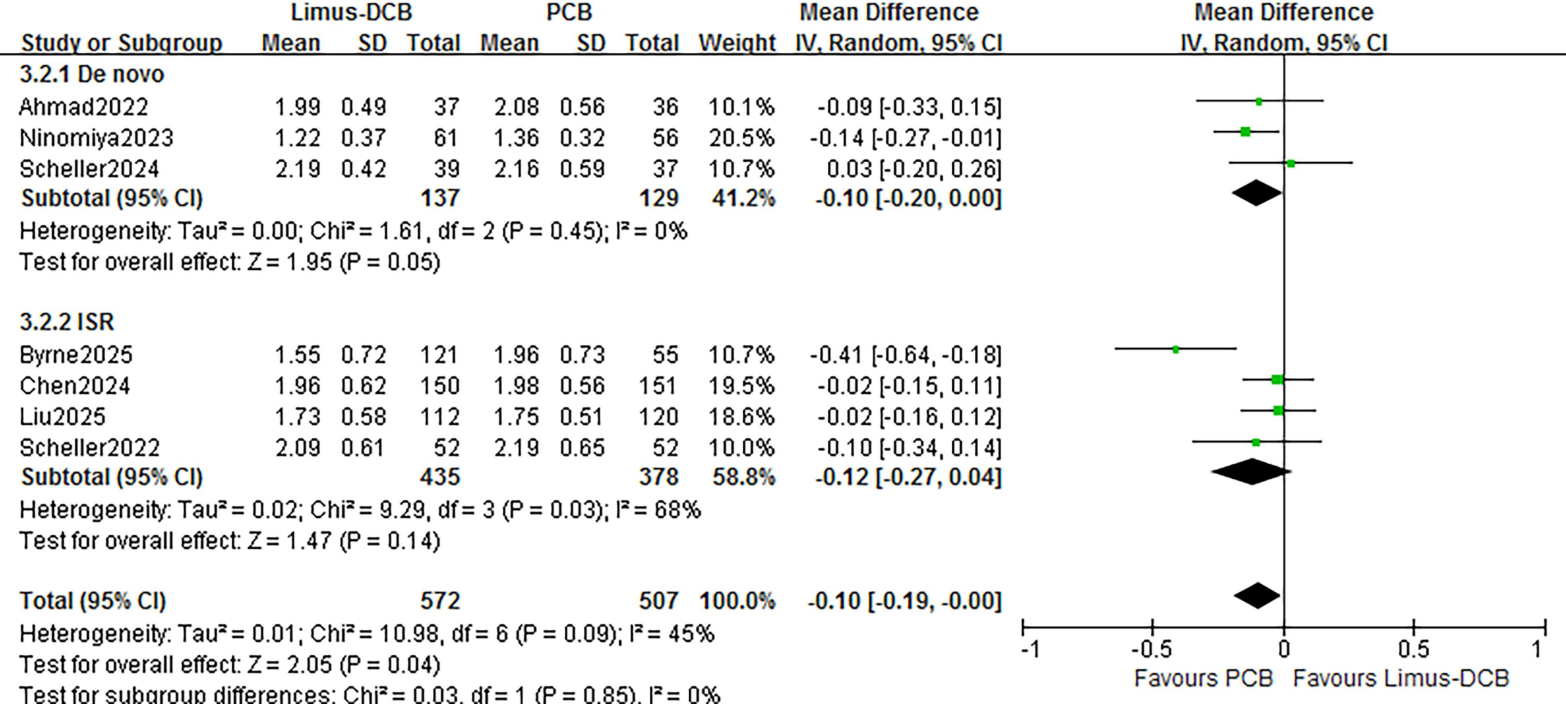
Forest plot of in-lesion MLD: limus-DCB versus PCB.

**Figure 5:**
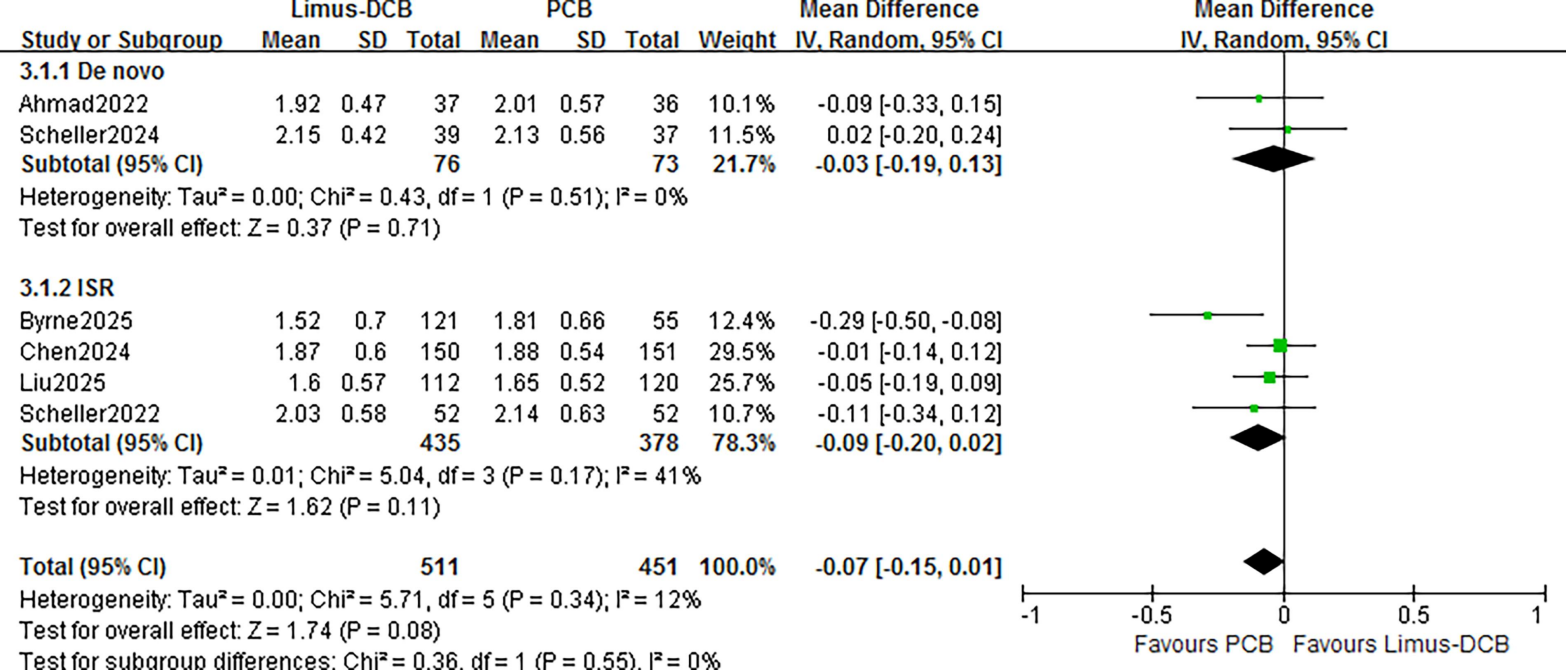
Forest plot of in-segment MLD: limus-DCB versus PCB.

**Figure 6:**
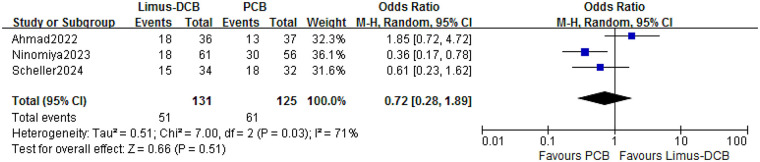
Forest plot of LLE: limus-DCB versus PCB.

**Figure 7:**
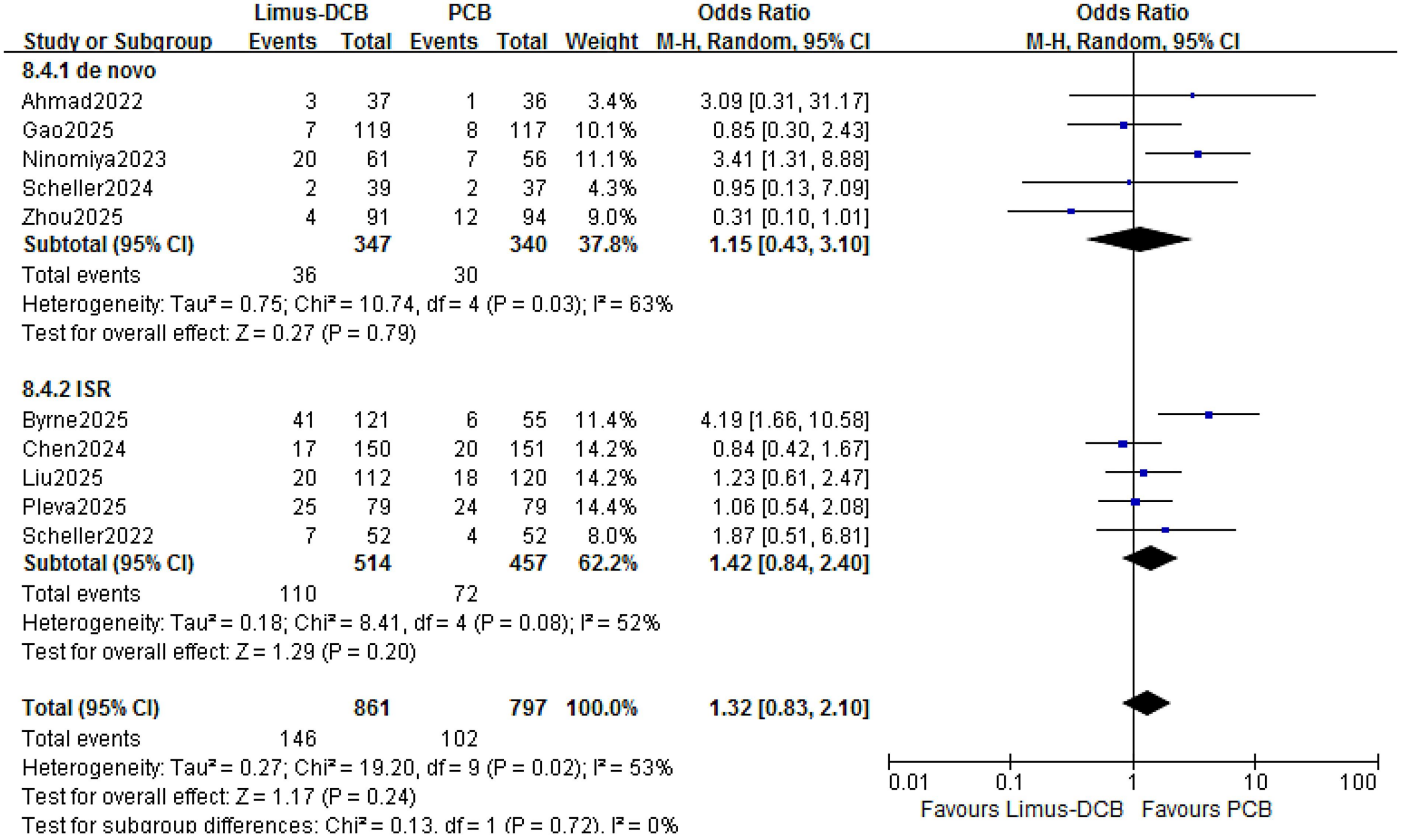
Forest plot of binary restenosis rate: limus-DCB versus PCB.

For in-segment LLL, the Limus-DCB group showed marginally greater loss compared with the PCB group (MD = 0.10 mm, 95% CI 0.00–0.19, *τ*^2^ = 0.01, 95% PI −0.16 to 0.36, *P* = 0.04, *I*^2^ = 68%). This difference was primarily observed in the *de novo* lesion subgroup (Figure 2). For in-lesion LLL, there was no significant difference between the two groups (MD = 0.11 mm, 95% CI −0.03 to 0.25, *τ*^2^ = 0.03, 95% PI −0.35 to 0.57, *P* = 0.12, *I*^2^ = 83%) with no significant interaction between both subgroups (Pinteraction = 0.97) (Figure 3). Regarding in-lesion MLD, the Limus-DCB group was associated with a slightly smaller MLD compared with the PCB group [MD = −0.10, 95% CI (−0.19, −0.00), *P* = 0.04, *I*^2^ = 45%] (Figure 4), while there was no significant difference between the two groups for in-segment MLD (Figure 5). There was no statistically significant difference in the rate of LLE between the two groups based on three *de novo* lesion studies [OR = 0.72, 95% CI (0.28, 1.89), *P* = 0.51, *I*^2^ = 71%](Figure 6). Similarly, no significant difference was observed in the binary restenosis rate (preferentially using in-segment data) between the two groups (OR = 1.32, 95% CI 0.83–2.10, *τ*^2^ = 0.27, 95% PI 0.36–4.80, *P* = 0.24, *I*^2^ = 53%)(Figure 7). Subgroup analysis showed no statistically significant differences in the binary restenosis rate for in-segment, in-lesion, SCB, or BCB (Table 3). Subgroup analysis showed that for in-segment LLL, SCB was associated with slightly greater LLL compared with PCB [MD = 0.09, 95% CI (0.00–0.18), *P* = 0.04]. For other comparisons (BCB for LLL, and SCB/BCB for MLD), no statistically significant differences were observed (Table 4).

**Table 3:** Subgroup analysis of the binary restenosis rate.

Subgroup	Included studies	Heterogeneity test		Meta-analysis results	
		*P*	*I*^2^(%)	OR (95% CI)	P
In-Segment	6 (6, 14–18)	0.02	63	1.18 (0.66, 2.12)	0.57
In-Lesion	6 (11, 13, 15, 16, 18, 19)	0.04	57	1.71 (0.78, 3.75)	0.18
SCB	8 (6, 11–14, 17–19)	0.13	38	1.21 (0.74, 1.96)	0.45
BCB	2 (15, 16)	0.006	87	1.82 (0.37, 8.87)	0.46

**Table 4:** Subgroup analysis of SCB and BCB.

Outcome measures	Subgroup	Included studies	Heterogeneity test		Meta-analysis results	
			*P*	*I^2^*(%)	OR (95% CI)	P
In-Segment LLL	SCB	6 (6, 11–14, 18)	0.12	43	0.09 (0.00, 0.18)	0.04
	BCB	2 (15, 16)	0.0005	92	0.15 (−0.20, 0.50)	0.40
In-Lesion LLL	SCB	5 (11–13, 18, 19)	0.00008	79	0.09 (−0.08, 0.26)	0.30
	BCB	2 (15, 16)	<0.00001	94	0.17 (−0.23, 0.57)	0.41
In-Segment MLD	SCB	4 (6, 11, 13, 18)	0.87	0	−0.05 (−0.15, 0.04)	0.28
	BCB	2 (15, 16)	0.03	79	−0.08 (−0.19, 0.03)	0.13
In-Lesion MLD	SCB	5 (6, 11–13, 18)	0.65	0	−0.08 (−0.15, 0.00)	0.06
	BCB	2 (15, 16)	0.004	88	−0.20 (−0.58, 0.18)	0.30

Sensitivity analysis using a fixed-effects model showed consistent results for in-segment LLL and in-lesion MLD. However, for in-lesion LLL and binary restenosis rate, the fixed-effects model yielded statistically significant differences favoring PCB, whereas the random-effects model did not. This discrepancy may be attributed to the substantial heterogeneity (*I*^2^ = 55% and 57%, respectively), for which the random-effects model provides more conservative estimates. Given the presence of heterogeneity, the random-effects model results were considered primary.

#### 3.3.2 Clinical outcome indicators

Meta-analysis using a random-effects model showed no statistically significant differences between the two groups for any of the clinical and procedural outcomes, including clinical procedural success, MACE, all-cause mortality, TLF, TLR, cardiac mortality, MI, TVMI, and thrombosis, with no important heterogeneity detected (*I*^2^ = 0%–30% for most outcomes) (Table 5). Using a fixed-effects model did not change the results.

**Table 5:** Meta-analysis of clinical outcome indicators.

Outcome measures	Included studies	Heterogeneity test		Meta-analysis results	
		*P*	*I^2^*(%)	OR (95% CI)	P
Procedural success	5 (6, 12, 15, 17, 18)	0.68	0	0.66 (0.16, 2.68)	0.56
MACE	8 (6, 11, 13–15, 17–19)	0.19	30	0.94 (0.65, 1.37)	0.76
All-cause mortality	8 (6, 13–19)	0.66	0	0.82 (0.34, 2.00)	0.66
TLF	7 (6, 12, 14–17, 19)	0.41	2	1.13 (0.80, 1.61)	0.49
TLR	8 (6, 11, 12, 14, 15, 17–19)	0.50	0	1.14 (0.79, 1.65)	0.49
Cardiac mortality	6 (6, 13–16, 18)	0.61	0	0.73 (0.21, 2.52)	0.61
MI	7 (6, 12, 14, 15, 17–19)	0.64	0	0.99 (0.40, 2.43)	0.98
TVMI	5 (6, 12, 15–17)	0.70	0	0.71 (0.27, 1.91)	0.50
Thrombosis	4 (12, 14, 15, 18)	0.65	0	1.03 (0.24, 4.51)	0.97

MACE, major adverse cardiovascular events; TLF, target lesion failure; TLR, target lesion revascularization; MI, myocardial infarction; TVMI, target vessel myocardial infarction.

**Figure 8:**
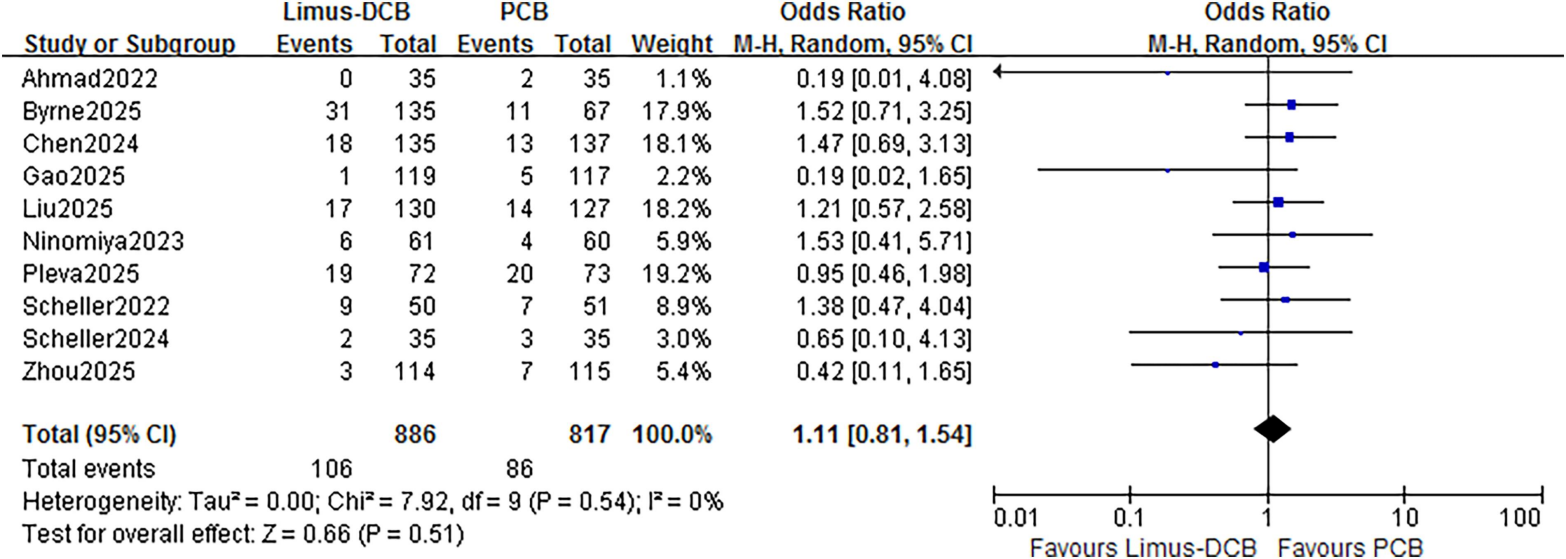
Forest plot of uniform definition of TLF: limus-DCB versus PCB.

Given the heterogeneity in MACE definitions across the included studies, we additionally performed a *post-hoc* analysis using a uniform definition of TLF—comprising cardiac death, TVMI, and TLR—as the primary composite endpoint, regardless of the definitions used in the original studies. Using this uniform definition, meta-analysis including all 10 studies similarly demonstrated no significant difference between the two groups (OR = 1.11, 95% CI 0.81–1.54, *I*^2^ = 0%) (Figure 8).

### 3.4 Publication bias assessment

**Figure 9:**
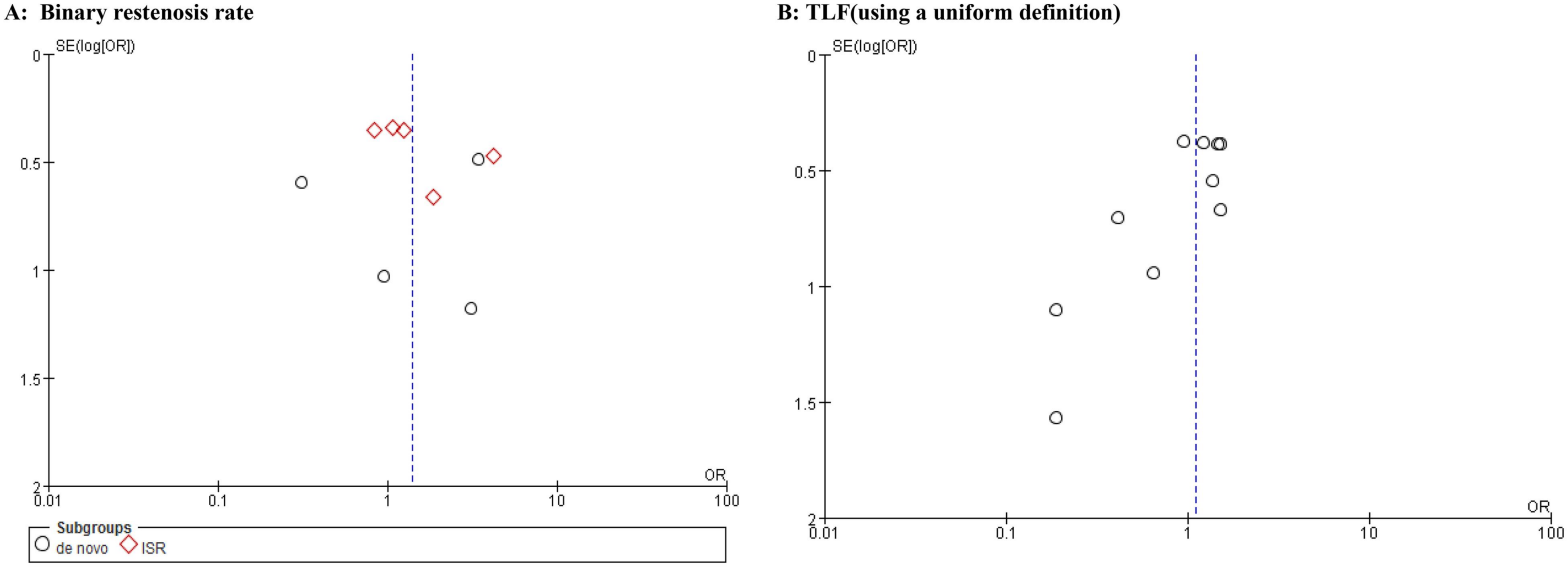
Funnel plot of binary restenosis rate and TLF (using a uniform definition) for publication bias assessment.

Given the limited number of included studies, the funnel plots should be interpreted with caution. The funnel plot for TLF (using a uniform definition) appeared roughly symmetrical around the pooled estimate; however, funnel plot interpretation is limited by the small number of studies, and this visual pattern should not be overinterpreted as definitive evidence against publication bias. In contrast, the funnel plot for binary restenosis rate showed asymmetry, with no studies falling in the OR > 1 region, indicating possible small-study effects for binary restenosis. (Figure 9).

### 3.5 Certainty of evidence

The certainty of evidence was assessed using the GRADE approach (21, 22). A summary of the GRADE evidence assessment is presented in Table 6.

**Table 6:** Summary of findings: limus-DCB versus PCB for coronary artery disease.

**Outcome (follow-up)**	**No. of participants (studies** ^a^ **)**	**Relative effect (95% CI)**	**Anticipated absolute effects (PCB)**	**Limus-DCB (95% CI)**	**Certainty**	**What happens**
In-segment LLL (6–12 mo) (mm, lower better)	1,237 vessels (8 studies)	MD 0.10 higher (0.00 to 0.19)	Mean LLL in PCB groups was 0.26 mm (range 0.01 to 0.62 mm)	0.10 higher (0.00 to 0.19)	MODERATE	Limus-DCB is associated with slightly greater LLL
In-lesion LLL (6–9 mo) (mm, lower better)	1,083 vessels (7 studies)	MD 0.11 higher (−0.03 to 0.25)	Mean LLL in PCB groups was 0.22 mm (range 0.00 to 0.31 mm)	0.11 higher (−0.03 to 0.25)	LOW	Evidence is uncertain; there may be little or no difference
In-lesion MLD (6–9 mo) (mm, higher better)	1,079 vessels (7 studies)	MD 0.10 lower (−0.19 to 0.00)	Mean MLD was 1.90 mm (range 1.36 to 2.19 mm)	0.10 lower (−0.19 to 0.00)	MODERATE	Limus-DCB is associated with slightly smaller minimal lumen diameter
Binary restenosis rate (6–9 mo)	1,658 vessels (10 studies)	OR 1.32 (0.83 to 2.10)	117 per 1,000^b^	154 per 1,000 (97 to 246)	MODERATE	May be little or no difference between groups
MACE (9–12 mo)	1,380 patients (8 studies)	OR 0.94 (0.65 to 1.37)	142 per 1,000	133 per 1,000 (92 to 195)	MODERATE	Limus-DCB likely results in little or no difference in MACE
TLF (9–12 mo)	1,462 patients (7 studies)	OR 1.13 (0.80 to 1.61)	95 per 1,000	107 per 1,000 (76 to 153)	MODERATE	Limus-DCB likely results in little or no difference in TLF
TLR (9–12 mo)	1,501 patients (9 studies)	OR 1.14 (0.79 to 1.65)	80 per 1,000	91 per 1,000 (63 to 132)	MODERATE	Limus-DCB likely results in little or no difference in TLR
All-cause mortality (9–12 mo)	1,703 patients (10 studies)	OR 0.82 (0.34 to 2.00)	12 per 1,000	10 per 1,000 (4 to 24)	LOW	Limus-DCB may result in little or no difference in all-cause mortality, but the evidence is uncertain due to very few events and a wide confidence interval that includes both potential benefit and harm
MI (9–12 mo)	1,431 patients (8 studies)	OR 0.99 (0.40 to 2.43)	17 per 1,000	17 per 1,000 (7 to 41)	LOW	Limus-DCB may result in little or no difference in MI, but the evidence is uncertain due to very few events and a wide confidence interval that includes both potential benefit and harm

a Include studies with no events in either arm.

b Based on median control group risk.

For angiographic outcomes, the certainty of evidence ranged from moderate to low. In-segment LLL was rated as MODERATE certainty, downgraded one level for serious inconsistency (*I*^2^ = 68%), as the 95% prediction interval (−0.16 to 0.36 mm) crossed zero, indicating that the true effect in future studies could differ substantially from the pooled estimate. In-lesion MLD was also rated as MODERATE certainty, downgraded one level for imprecision because the upper bound of the confidence interval touched the null value (−0.19 to 0.00). In-lesion LLL was rated as LOW certainty due to very serious inconsistency (*I*^2^ = 83%) and serious imprecision (wide confidence interval crossing zero), indicating that the true effect may be substantially different from the pooled estimate. Binary restenosis rate was rated as MODERATE certainty, downgraded for publication bias (asymmetric funnel plot suggestive of small-study effects) and moderate inconsistency (*I*^2^ = 53%).

For clinical outcomes, the certainty of evidence was MODERATE across most endpoints, including MACE, TLF and TLR. Although all included trials were open-label, which may introduce performance bias, most studies used blinded independent clinical event committees to adjudicate outcomes, mitigating detection bias. Nevertheless, we downgraded one level for risk of bias. No downgrading was applied for inconsistency (*I*^2^ = 0%–30% for most clinical outcomes). For all-cause mortality (OR 0.82, 95% CI 0.34–2.00; 23 events) and MI (OR 0.99, 95% CI 0.40–2.43; 22 events), the confidence intervals were wide and crossed the null, indicating serious imprecision due to the very low number of events. We therefore downgraded the certainty of evidence by one level to LOW for both outcomes.

Overall, while the angiographic evidence suggests modest differences favoring PCB, the clinical evidence—moderate certainty for MACE, TLF, and TLR, but low certainty for mortality and MI—indicates that no statistically significant differences were detected in the composite short-term clinical endpoints, although uncertainty remains for rare outcomes.

## 4 Discussion

Several previous studies have investigated the comparative efficacy of Limus-DCB vs. PCB for coronary artery disease, but the conclusions have been inconsistent. Chen et al. (15) found that when treating ISR, coronary angiography at 9 months post-procedure suggested that BCB was non-inferior to PCB regarding in-segment LLL (0.25 ± 0.40 mm in 150 vessels vs. 0.27 ± 0.39 mm in 151 vessels, *P* = 0.670). These results are consistent with the meta-analysis conclusions by Shin et al. (23) However, the study by Ninomiya et al. (12) found that when treating *de novo* lesions, SCB resulted in greater LLL (0.32 ± 0.47 mm in 61 vessels vs. 0.00 ± 0.32 mm in 56 vessels, *P* < 0.0001). The present meta-analysis builds upon the design of the study by Ramy et al. (24) but incorporates four recently published RCTs (6, 14, 16, 19). Unlike recent reviews (25) that have focused specifically on ISR lesions, our analysis includes both *de novo* and ISR lesions, offering a broader perspective on the comparative efficacy of Limus-DCB vs. PCB across different coronary lesion subsets. Compared with previous meta-analysis (24), our updated analysis suggests that the angiographic advantage of PCB over Limus-DCB may be smaller than previously estimated (MD for in-segment LLL: 0.10 mm, 95% CI 0.00–0.19, with the lower bound abutting the null value; MD for in-lesion MLD: −0.10 mm, 95% CI −0.19–0.00, with the upper bound abutting the null value), indicating that the differences between the two devices may become less pronounced as more evidence accumulates. Subgroup analysis by balloon drug class showed that SCB was associated with slightly greater in-segment LLL compared with PCB (MD = 0.09, 95% CI 0.00–0.18, *P* = 0.04), whereas BCB did not differ significantly from PCB in angiographic outcomes, although this finding is limited by the small number of BCB studies (*n* = 2) and substantial heterogeneity. Regarding clinical outcomes, Limus-DCB and PCB were similar with respect to MACE, TLF, and TLR. It is important to note that the observed angiographic differences were small in magnitude (0.10 mm for in-segment LLL and −0.10 mm for in-lesion MLD), and their clinical relevance remains uncertain given the absence of significant differences in clinical outcomes. The moderate-certainty clinical evidence for the primary composite endpoints (MACE, TLF, and TLR) suggests that, although residual bias cannot be fully excluded, the finding of no clinically meaningful differences is supported with moderate certainty. These small angiographic differences may not translate into clinically meaningful benefits or harms for patients, and the comparable clinical outcomes suggest that both devices are acceptable treatment options from a patient-centered perspective.

Significant differences exist between paclitaxel and limus agents regarding their anti-proliferative mechanisms and pharmacokinetics. Paclitaxel is a cytotoxic diterpenoid that binds to the β-subunit of tubulin, irreversibly arresting the cell cycle at the G2/M phase and inducing apoptosis (11, 26–28). Its high lipophilicity (logP 3.96) allows for rapid tissue uptake and prolonged retention, with drug concentrations persisting in tissues for several weeks after a single application (29). This property ensures that paclitaxel produces a durable anti-proliferative effect even after brief balloon inflation (30–60 s) (30). Therefore, paclitaxel is highly suitable for DCB. However, its cytotoxic nature may delay endothelial healing and potentially increase inflammatory responses in certain situations (28). In contrast, sirolimus is a cytostatic macrolide that reversibly inhibits the mTOR pathway by binding to FKBP12, reversibly arresting the cell cycle at the G1 phase (31, 32). It has lower lipophilicity (logP 2.5) and a shorter tissue half-life, requiring advanced delivery systems (e.g., phospholipid carriers, micro-reservoir formulations) for effective tissue transfer and retention (33–36). Preclinical studies suggest that, compared with paclitaxel, sirolimus promotes faster re-endothelialization and reduces inflammation, indicating a potentially superior healing profile (28). However, our meta-analysis showed that SCB was associated with greater LLL, which contrasts with earlier DES trials (37, 38) where sirolimus was superior to paclitaxel. This discrepancy may be explained by insufficient tissue retention of sirolimus from balloon delivery, as the brief inflation time may not allow adequate drug transfer and retention, increasing the risk of late restenosis.

Biolimus, a limus derivative with approximately 10-fold higher lipophilicity than sirolimus (39), might be expected to perform better. However, our subgroup analysis did not demonstrate superiority of BCB over PCB. This may be attributed to the limited number of BCB studies (*n* = 2) with substantial heterogeneity for angiographic outcomes, different device platforms (BioAscend JWMS vs. Biosensors Europe BCB), and potential differences in patient populations. Furthermore, like sirolimus, biolimus binds reversibly to FKBP12 (40). This reversibility suggests that once the local drug concentration falls below an effective threshold, its anti-proliferative effect may diminish. In the context of a single, brief balloon inflation, the requirement for sustained target inhibition could represent a pharmacological limitation for the BCB. In contrast, paclitaxel's irreversible action may offer more durable efficacy, even after transient exposure. Therefore, even with improved lipophilicity, the reversible nature of the BCB might partly explain why it did not demonstrate superior angiographic outcomes in this study. Larger head-to-head trials with standardized platforms and longer follow-up are needed to further evaluate BCB vs. PCB.

The substantial heterogeneity observed for in-segment LLL (*I*^2^ = 68%), in-lesion LLL (*I*^2^ = 83%), and LLE (*I*^2^ = 71%) warrants further discussion. Several factors may contribute to this heterogeneity. First, the included studies used different Limus-DCB platforms with varying excipient technologies, including SeQuent Neo SCB (B. Braun), MagicTouch SCB (Concept Medical), Revita Medical Technology SCB, BioAscend JWMS BCB, and Biosensors Europe BCB. Differences in drug transfer efficiency and tissue retention across these platforms may affect angiographic outcomes. Second, the studies enrolled patients with different lesion types (*de novo* vs. ISR) and reference vessel diameters (ranging from 2.0 mm to 5.0 mm). Notably, the heterogeneity for in-lesion LLL was substantially higher in the ISR subgroup (*I*^2^ = 87%) compared with the *de novo* subgroup (*I*^2^ = 55%). The TRANSFORM-I trial Ninomiya et al. (12) and the REFORM trial Byrne et al. (16) were major contributors to this heterogeneity, both using different platforms (MagicTouch SCB and Biosensors Europe BCB, respectively). Third, angiographic follow-up durations varied from 6 to 12 months across studies for LLL outcomes, which may contribute to heterogeneity. However, for LLE, all three included studies had consistent 6 month follow-up, so follow-up duration does not explain the heterogeneity in LLE. Fourth, for LLE (*I*^2^ = 71%), the analysis was limited to three *de novo* lesion studies. The heterogeneity may reflect variability in lesion preparation techniques (e.g., cutting balloons, scoring balloons) and vessel responses across these trials.

The calculated 95% prediction intervals for in-segment LLL (−0.16 to 0.36 mm) and binary restenosis rate (0.36–4.80) indicate substantial uncertainty. For in-segment LLL, the prediction interval crosses zero, meaning that a future study could find either a slight advantage for PCB or a slight advantage for Limus-DCB. For binary restenosis rate, the wide prediction interval (0.36–4.80) suggests that the true effect in a new setting could range from a large protective effect of PCB to a substantial excess risk with Limus-DCB. These wide intervals reflect the considerable heterogeneity across studies and underscore the need for caution when generalizing the pooled estimates. These factors should be considered when interpreting the pooled results, and the random-effects model was appropriately chosen to account for this heterogeneity.

Several limitations of this study warrant consideration. First, regarding study design, all included trials were open-label, which may introduce performance bias. Second, concerning data quality and completeness, not all patients completed angiographic follow-up; although most studies used intention-to-treat analysis for clinical outcomes, the available-case analysis for angiographic endpoints may introduce attrition bias. Furthermore, the absence of intravascular imaging (e.g., IVUS, OCT) support for vessel measurements may affect the accuracy of quantitative coronary angiography. Third, all trials were conducted in Asia and Europe, lacking data from regions such as Africa and the Americas, which may limit the generalizability of our findings to broader populations. Fourth, angiographic outcomes were vessel-level, while clinical outcomes were patient-level. Some studies may have included multiple vessels from the same patient, introducing clustering effects that we could not adjust for due to the lack of individual patient data. This may have led to underestimated standard errors for vessel-level outcomes. Fifth, substantial heterogeneity existed across studies in terms of lesion types (*de novo* vs. ISR), investigational DCB platforms (five different Limus-DCB devices with varying excipient technologies), and follow-up durations (6–12 months). Notably, the number of BCB studies was small (*n* = 2), precluding robust subgroup conclusions for BCB. Sixth, the maximum clinical follow-up among the included studies was only 12 months. Longer-term outcomes beyond one year, including very late restenosis, late catch-up phenomenon, and very late stent thrombosis, remain unknown. Seventh, we did not systematically search trial registries such as ClinicalTrials.gov or the WHO ICTRP, which may have resulted in the omission of unpublished or ongoing trials. Eighth, the Hartung-Knapp adjustment was not implemented.

## 5 Conclusion

Current evidence suggests that Limus-DCB is associated with less favorable angiographic outcomes than PCB in the treatment of coronary artery disease, while no statistically significant short-term clinical differences were detected between the two groups. However, the observed angiographic differences were small, and their clinical relevance remains uncertain. Given the limitations in the quantity and quality of the included studies, these findings should be interpreted with caution. Future large-scale, high-quality RCTs with extended follow-up are needed to further evaluate the comparative efficacy of BCB and next-generation Limus-DCB, particularly using standardized imaging endpoints and individual patient data to confirm these findings.

## Data Availability

The original contributions presented in the study are included in the article/Supplementary Material, further inquiries can be directed to the corresponding author.
