# Supplementary material for: A meta-analysis of the efficacy of limus-coated balloons vs. paclitaxel-coated balloons for coronary artery disease
Source: Front Cardiovasc Med. 2026 Jul 14;13:1872397. doi: 10.3389/fcvm.2026.1872397 (PMC13408383; doi:10.3389/fcvm.2026.1872397)
Supplement: Supplementary file 2 [file Table1.doc]

**Supplemental Table 1. Definitions of outcome measures reported in the included studies.**

**Study: Liu2025**

**MACE:** Not directly defined. Used PoCE (all-cause death, all MI, any revascularization).

**TLF:** Cardiac death, target vessel MI, and clinically-driven TLR.  **TLR:** Clinically-driven target lesion revascularization.

**Cardiac mortality:** Not defined. **MI:** Not defined. **TVMI:** Not defined，reported as "target vessel MI."

**Procedural Success:** Lesion success (residual stenosis ≤30%, TIMI 3 flow, no type C or worse dissection) without MACE (death, MI, and target

lesion revascularization) before discharge.

**Study: Scheller2024**

**MACE:** Cardiac death, target vessel MI, or clinically-driven TLR. **TLR:** Per ARC definitions(2007). **Cardiac mortality:** Per ARC definitions(2007).

**Thrombosis:** Per ARC definitions(2007).

**Study: Ninomiya2023**

**TLF:** Not directly defined. Used DoCE: Cardiac death, target vessel MI, and TLR.

**TLR:** Clinically and/or physiologically-indicated target lesion revascularization. **Cardiac mortality: Not defined.**

**MI:** Per fourth universal definition.  **TVMI:** Per fourth universal definition. **Thrombosis:** Not defined.

**Procedural Success:** Final in-lesion residual stenosis of <30% without the occurrence of TLF during the index procedure hospital stay.

**Study: Ahmad2022**

**MACE:** Cardiac death, target vessel MI, or clinically-driven TLR.  **TLR:** Per ARC definitions(2007).

**Cardiac mortality:** Per ARC definitions(2007).  **MI:** Not defined. **TVMI:** Per ARC definitions(2007). **Thrombosis:** Per ARC definitions.

**Study: Pleva2025**

**MACE:** Cardiovascular death, any MI, or TLR. **TLF:** Cardiovascular death, target vessel MI, or ischemia-driven TLR. **TLR:** Per ARC definitions(2018)

**Cardiac mortality:** Per ARC definitions(2018). **MI:** Per ESC fourth universal definition.  **Thrombosis:** Per ARC definitions(2018).

**Study: Chen2024**

**MACE:** Not directly defined. Used PoCE (all-cause death, all MI, any revascularization).

**TLF:** Cardiac death, target vessel MI, and clinically-driven TLR.  **TLR:** Not defined. **MI:** Not defined. **TVMI:** Not defined, reported as "target vessel MI."

**Thrombosis:** Not defined.

**Procedural Success:** Lesion success (residual stenosis ≤30%, TIMI 3 flow, no type C or worse dissection) with absence of death, MI, and TLR before discharge.

**Study: Byrne2025**

**TLF:** Cardiovascular death, any target vessel MI, CABG, and clinically-driven TLR. **Cardiac mortality:** Not defined.

**TVMI:** Not defined, reported as "target vessel MI."

**Study: Zhou2025**

**MACE:** Not directly defined. Used PoCE (all-cause death, MI, ischemia-driven revascularization). **TLF:** Not directly defined. Used DoCE (cardiac death, target vessel-related MI, ischemia-driven TLR). **TLR:** Ischemia-driven target lesion revascularization. **Cardiac mortality:** Not defined.

**MI:** Not defined. **TVMI:** Not defined,reported as "target vessel-related MI."

**Procedural Success:** Lesion success (residual stenosis ≤30%, TIMI 3 flow) with absence of in-hospital cardiac death, target vessel-related MI, and TLR.

**Study: Scheller2022**

**MACE:** Cardiac death, target vessel MI, or clinically-driven TLR.  **TLR:** Clinically-driven target lesion revascularization, per ARC definitions(2007).

**Cardiac mortality:** Per ARC definitions(2007). **MI:** Not defined.

**Procedural Success:** Final stenosis <30%, TIMI 3 flow, no flow-limiting dissection, and absence of in-hospital MACE.

**Study: Gao2025**

**MACE:** All-cause mortality, MI, and revascularization. **TLF:** Cardiac death, target vessel-related MI, and TLR.

**TLR:** A new intervention (surgical or percutaneous) to treat the target lesion, due to any evidenced ischemia (anatomical or functional) related to the restenosis of the target lesion. **MI:** Not defined.
