# Supplementary material for: A meta-analysis of the efficacy of limus-coated balloons vs. paclitaxel-coated balloons for coronary artery disease
Source: Front Cardiovasc Med. 2026 Jul 14;13:1872397. doi: 10.3389/fcvm.2026.1872397 (PMC13408383; doi:10.3389/fcvm.2026.1872397)
Supplement: Supplementary file 3 [file Table2.doc]

### Supplemental Table 2. Search strategies.

**PubMed Search Strategy:**

#1 "Angioplasty, Balloon, Coronary"[mh]

#2 "Drug-Eluting Balloon"[tiab]

#3 "Drug-Coated Balloon"[tiab]

#4 "DEB"[tiab]

#5 "DCB"[tiab]

#6 "*eluting Balloon"[tiab]

#7 "*coated Balloon"[tiab]

#8 #1 OR #2 OR #3 OR #4 OR #5 OR #6 OR #7

#9 "Sirolimus"[Mesh]

#10 "Sirolimus*"[tiab]

#11 "Rapamycin"[tiab]

#12 "Rapamune"[tiab]

# 13 "*limus"[tiab]

#14 "biolimus*"[tiab]

#15 "rapalog*"[tiab]

#16 "umirolimus"[Supplementary Concept]

#17 “mTOR inhibitor*"[tiab]

#18 #9 OR #10 OR #11 OR #12 OR #13 OR #14 OR #15 OR #16 OR #17

#19 "paclitaxel"[Mesh]

#20 "paclitaxel*"[tiab]

#21 "Anzatax"[tiab]

#22 "Taxol"[tiab]

#23 "Paxene"[tiab]

#24 "Praxel"[tiab]

#25 "Onxol"[tiab]

#26 #19 OR #20 OR #21 OR #22 OR #23 OR #24 OR #25

#27 "Randomized Controlled Trial"[pt]

#28 "Controlled Clinical Trial"[pt]

#29 "randomised"[tiab]

#30 "randomly"[tiab]

#31 "trial"[tiab]

#32 "groups"[tiab]

#33 "placebo"[tiab]

#34 "clinical trials as topic"[mesh:noexp]

#35 "animals"[mh]

#36 "humans"[mh]

#37 #35 NOT #36

#38 #27 OR #28 OR #29 OR #30 OR #31 OR #32 OR #33 OR #34 NOT #37

#39 #8 AND #18 AND #26 AND #38

**Embase Search Strategy:**

#1 'transluminal coronary angioplasty'/exp

#2 'Drug-Coated Balloon'/exp

#3 'Drug-Eluting Balloon':ab,ti

#4 'DEB':ab,ti

#5 'DCB':ab,ti

#6 'eluting Balloon':ab,ti

#7 'coated Balloon':ab,ti

#8 #1 OR #2 OR #3 OR #4 OR #5 OR #6 OR #7

#9 'Sirolimus'/exp

#10 'Sirolimus*':ab,ti

#11 'Rapamycin':ab,ti

#12 'Rapamune':ab,ti

#13 'limus':ab,ti

#14 'biolimus':ab,ti

#15 'umirolimus':ab,ti

#16 'rapalog*':ab,ti

#17 'mTOR inhibitor*':ab,ti

#18 #9 OR #10 OR #11 OR #12 OR #13 OR #14 OR #15 OR #16 OR #17

#19 'paclitaxel'/exp

#20 'paclitaxel*':ab,ti

#21 'Anzatax':ab,ti

#22 'Taxol':ab,ti

#23 'Paxene':ab,ti

#24 'Praxel':ab,ti

#25 'Onxol':ab,ti

#26 #19 OR #20 OR #21 OR #22 OR #23 OR #24 OR #25

#27 'randomised controlled trial'/exp

#28 'randomised':ab,ti

#29 'randomly':ab,ti

#30 'placebo':ab,ti

#31 #27 OR #28 OR #29 OR #30

#32 #8 AND #18 AND #26 AND #31

**Cochrane Library Search Strategy:**

#1 MeSH descriptor: [Angioplasty, Balloon, Coronary] explode all trees

#2 (Drug-Eluting Balloon):ti,ab,kw

#3 (Drug-Coated Balloon):ti,ab,kw

#4 (DEB):ti,ab,kw

#5 (DCB):ti,ab,kw

#6 (*eluting balloon):ti,ab,kw

#7 (*coated balloon):ti,ab,kw

#8 #1 OR #2 OR #3 OR #4 OR #5 OR #6 OR #7

#9 MeSH descriptor: [Sirolimus] explode all trees

#10 (Sirolimus*):ti,ab,kw

#11 (Rapamycin):ti,ab,kw

#12 (Rapamune):ti,ab,kw

#13 (*limus):ti,ab,kw

#14 (biolimus*):ti,ab,kw

#15 (umirolimus):ti,ab,kw

#16 (rapalog*):ti,ab,kw

#17 (mTOR inhibitor*):ti,ab,kw

#18 #9 OR #10 OR #11 OR #12 OR #13 OR #14 OR #15 OR #16 OR #17

#19 MeSH descriptor: [Paclitaxel] explode all trees

#20 (Paclitaxel*):ti,ab,kw

#21 (Anzatax):ti,ab,kw

#22 (Taxol):ti,ab,kw

#23 (Paxene):ti,ab,kw

#24 (Praxel):ti,ab,kw

#25 (Onxol):ti,ab,kw

#26 #19 OR #20 OR #21 OR #22 OR #23 OR #24 OR #25

#27 #8 AND #18 AND #26

**Web of Science Search Strategy:**

#1 TS="Coronary balloon angioplasty" OR "Drug-Eluting Balloon" OR "Drug-Coated Balloon" OR DEB OR DCB OR "eluting balloon" OR "coated balloon"

#2 TS=Sirolimus* OR Rapamycin OR Rapamune OR limus OR biolimus* OR umirolimus OR rapalog* OR "mTOR inhibitor*"

#3 TS=Paclitaxel* OR Anzatax OR Taxol OR Paxene OR Praxel OR Onxol

#4 TS=Random* OR groups OR trial

#5 #1 AND #2 AND #3 AND #4
