# Supplementary material for: A meta-analysis of the efficacy of limus-coated balloons vs. paclitaxel-coated balloons for coronary artery disease
Source: Front Cardiovasc Med. 2026 Jul 14;13:1872397. doi: 10.3389/fcvm.2026.1872397 (PMC13408383; doi:10.3389/fcvm.2026.1872397)
Supplement: Supplementary file 4 [file Table3.doc]

### Supplemental Table 3. Full-text articles excluded with reasons.

| **Excluded study** | **Title** | **Reason for exclusion** |
| --- | --- | --- |
| Fitzgerald 2023 | A prospective, randomized, non-inferiority trial to determine the safety and efficacy of the Biolimus A9™ drug coated balloon for the treatment of in-stent restenosis: first-in-man trial (REFORM) | Conference abstract with subsequent full-text publication¹ |
| Alfonso 2015 | A Prospective Randomized Trial of Drug-Eluting Balloons Versus Everolimus-Eluting Stents in Patients With In-Stent Restenosis of Drug-Eluting Stents: The RIBS IV Randomized Clinical Trial | Stent vs. drug-coated balloon |
| Alfonso 2018 | 3-Year clinical follow-up of the RIBS IV clinical trial: a prospective randomized study of drug-eluting balloons versus everolimus-eluting stents in patients with in-stent restenosis in coronary arteries previously treated with drug-eluting stents | Stent vs. drug-coated balloon |
| Ali 2019 | Treatment of coronary drug-eluting stent restenosis by a sirolimus- or paclitaxel-coated balloon | Overlapping study population² |
| Wong 2018 | Comparison of drug-eluting stents and drug-coated balloon for the treatment of drug-eluting coronary stent restenosis: a randomized RESTORE trial | Stent vs. Drug Balloon |
| Ricardo 2022 | Sirolimus-eluting balloon with a micro-reservoir-based technology for the treatment of de novo coronary lesions- subgroup analysis of a prospective, multi-center, pivotal single-arm trial | Conference abstract; SCB vs. POBA (no PCB comparator) |
| Tobe 2024a³ | Changes of angiography-derived coronary microcirculatory resistance before and after drug-coated balloon treatment | Conference abstract; subanalysis of included study |
| Durand 2024 | Biolimus A9 DEB for the treatment of in-stent restenosis: 1-year outcomes of the REFORM trial | Conference abstract with subsequent full-text publication¹ |
| Pavel 2023 | Comparison of the efficacy of sirolimus and paclitaxel-eluting balloon catheters in the treatment of coronary in-stent restenosis: the TIS-2 study | Conference abstract with subsequent full-text publication¹ |
| Tobe 2024b³ | Quantitative coronary dissection volume after sirolimus or paclitaxel coated balloon and lumen changes at 6 months: serial OCT sub-study of TRANSFORM-trial | Conference abstract; subanalysis of included study |
| Ha 2026 | Paclitaxel versus sirolimus based balloon angioplasty for in-stent restenosis: a comparison of the AGENT IDE and SELUTION4ISR randomized pivotal controlled trials | SCB or PCB vs. POBA (no head-to-head comparison) |
| Gao 2024 | Drug-coated balloon angioplasty with rescue stenting versus intended stenting for the treatment of patients with de novo coronary artery lesions (REC-CAGEFREE I): an open-label, randomised, non-inferiority trial | Stent vs. drug-coated balloon |

Notes:

¹Full-text publication of the same trial was subsequently published and included in the meta-analysis where eligible.

²Ali 2019 was excluded because its patient population was included in the combined analysis of Scheller 2022(reference 18) to avoid double-counting.

³Tobe 2024 appears twice for two different conference abstracts;differentiated as Tobe 2024a and Tobe 2024b.
